# Dynamic Mechanical Analysis of Thermally Aged Fique Fabric-Reinforced Epoxy Composites

**DOI:** 10.3390/polym13224037

**Published:** 2021-11-22

**Authors:** Michelle Souza Oliveira, Fernanda Santos da Luz, Fabio da Costa Garcia Filho, Artur Camposo Pereira, Vinícius de Oliveira Aguiar, Henry Alonso Colorado Lopera, Sergio Neves Monteiro

**Affiliations:** 1 Department of Materials Science, Military Institute of Engineering—IME, Rio de Janeiro 22290-270, Brazil; oliveirasmichelle@ime.eb.br (M.S.O.); fernandaluz@ime.eb.br (F.S.d.L.); camposo@ime.eb.br (A.C.P.); 2 Department of Mechanical and Aerospace Engineering, University of California San Diego—UCSD, La Jolla, CA 92093, USA; fdacostagarciafilho@eng.ucsd.edu; 3 Department of Polymer Science and Technology, Institute of Macromolecules Professor Eloisa Mano—IMA, Rio de Janeiro 21941-598, Brazil; nviny_815@hotmail.com; 4 CCComposites Laboratory, Universidad de Antioquia—UdeA, Calle 70 n° 52-21, Medellin 050010, Colombia; henry.colorado@udea.udea.edu.co

**Keywords:** fique fabric, epoxy composite, DMA, accelerated aging, exponentially modified Gauss distribution

## Abstract

Dynamic mechanical analysis (DMA) is one of the most common methods employed to study a material’s viscoelastic properties. The effect of thermal aging on plain epoxy and a fique fabric-reinforced epoxy composite was investigated by comparing the mass loss, morphologies, and DMA properties of aged and unaged samples. In fact, thermal aging presents a big challenge for the high-temperature applications of natural fiber composites. In this work, both plain epoxy and fique fabric-reinforced epoxy composite were found to have different molecular mobility. This leads to distinct transition regions, with different changes in intensity caused by external loadings from time-aging. Three exponentially modified Gauss distribution functions (EMGs) were applied to loss factor curves of fique fabric-reinforced epoxy composite and plain epoxy, which allowed identifying three possible mobility ranges. From these results it was proposed that the thermal degradation behavior of natural fibers, especially fique fiber and their composites, might be assessed, based on their structural characteristics and mechanical properties.

## 1. Introduction

One of the most common methods used to measure a material’s viscoelastic properties is the dynamic mechanical analysis (DMA). Based on DMA it is possible to have information about the temperature- and time-dependent behavior [1,2], as well as aging [3,4,5], degradation [6], glass transition [7,8,9], degree of crosslinking [10,11,12], extent of phase mixing in blends [13,14], crystallinity [15], and interfacial adhesion [16,17,18], among others. Recently, the use of DMA as a characterization technique was applied to investigate mechanical properties of heterogeneous materials [19,20,21,22], spatial distribution of a material’s properties [23], and crack healing [24].

The importance of the accelerated aging studies has been recognized as a research method, which serves the purpose of determining the usability conditions and period of a product [25]. Natural fiber-reinforced polymer composites (NFRPC), as any other product for technical application, undergo gradual degradation with the passing of time. The main reasons for aging in NFRPC are the operating conditions and the usage and storage procedures of these materials. The property changes’ predictions of NFRPC are relevant to the security usage and reliability of the material [26,27]. 

It has been discussed that reinforcement of natural fibers in the thermoplastics and thermoset matrices offers several benefits such as having low cost, being ecofriendly renewable, and causing less damage to processing equipment compared to synthetic reinforcements [28]. This discussion also addresses the fact that natural fibers present relatively lower mechanical properties due to inherently weaker adhesion to polymers’ matrices when compared with synthetic fibers [28,29]. Other considerations for long-term sustainability and market acceptability of NFRPC include (1) energy and costs involved in the fiber supply chain; (2) a continuous and reliable source of natural fibers; (3) tax incentives for production; and (4) use in applications and (v) recycling [29,30].

The use of NFRPC is expected to grow in the future for specific applications, for example, use of light-weight natural fiber composites in ballistic armor [31,32,33]. In this case, thermal aging might play an important role in the armor expiration time. Indeed, aging affects the molecular mobility in both polymer matrix and natural fiber, which could accelerate the composite degradation under combined conditions of time and temperature [8]. 

**Figure 1:**
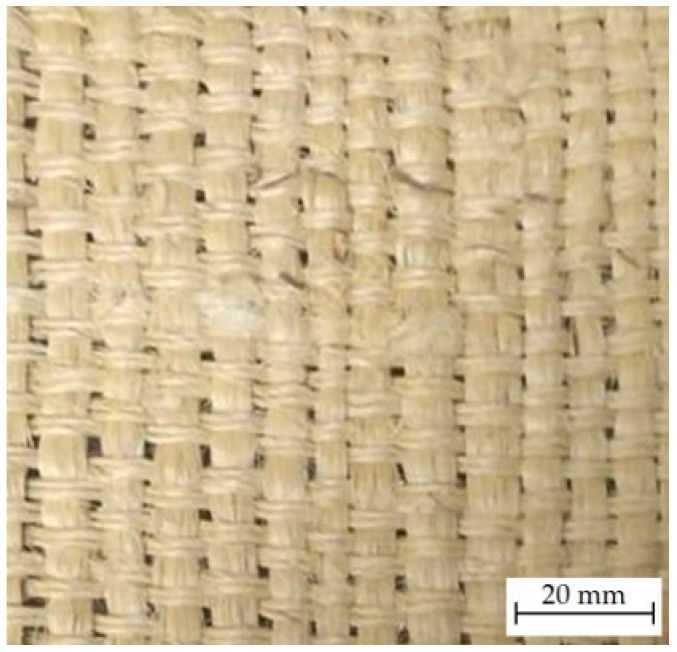
Fique fabric plain woven.

Among the lesser known natural fibers that have been investigated as reinforcement of polymer composites stands the fique fiber extracted from the South American plant *Furcraea andina* [34,35,36]. In particular, textiles made of fique fiber, as illustrated in Figure 1, were found to be a promising reinforcement for polymer matrix composites [20,22,37].

A recent investigation disclosed the superior ballistic performance of fique fabric as personal armor in comparison to worldwide-used synthetic aramid fabric [38,39,40]. A previous report on DMA results of polyester composites reinforced with up to 30 vol% of fique fabric [22] revealed a rise in viscoelastic stiffness and less mobility of the polyester chains. Moreover, a high attenuation of internal vibration and an increase in the glass transition temperature occurred with increasing the amount of fique fabric composites with up to 50 vol% of fique fabric [20], disclosing not only a superior ballistic performance compared to Kevlar™ but also economic and environmental advantages [39].

However, for successful application of these composites as ballistic armor their change under aging at a limit degradation temperature must be evaluated. Hence, the present work investigated the accelerated thermal aging of 40 vol% fique fabric-reinforced epoxy composite and the matrix itself, here named as plain epoxy (PE).

## 2. Materials and Methods

### 2.1. Materials

The fique fabric, Figure 1, was purchased in the market of Medellin, Colombia, by one of the authors of this work (H.A.C.L.). The fabric areal density, 859 g/cm^2^, and volume density, 1.53 g/cm^3^, were measured according to the corresponding standards in a previous article [40]. The matrix was the diglycidyl ether of bisphenol A (DGEBA) epoxy resin mixed with the triethylenetetramine (TETA) hardener in the stoichiometric proportion phr 13. Both DGEBA and TETA were supplied by Epoxyfiber, Rio de Janeiro, Brazil.

### 2.2. Composite Fabrication

The 40 vol% fique fabric–epoxy matrix composite was fabricated by placing the previously dried fabric piece as ply layers inside a steel mold, and DGEBA-TETA resin was also poured in. Laminated plates of 150 × 120 × 3.2 mm were made by the compression molding process and cured for 24 h at room temperature, inside the mold, under a 5-ton load. For DMA tests, these plates were cut into specimens, with dimensions of 50 × 13 × 3.2 mm^3^, in a Buehler Isomet cutoff machine (Lake Bluff, Lake County, IL, USA) using a diamond blade. Methods of accelerated thermal aging, as further described, were applied to these specimens.

### 2.3. Accelerated Aging

The fique fabric–epoxy composite and plain epoxy samples were subjected to an accelerated aging test performed in air chambers (Nova Instruments, Wakefield, MA, USA) at 170 °C for progressively increasing time lengths (0, 3, 5, and 10 days). The aging temperature of 170 °C was selected as the limit temperature before degradation in both the epoxy [41,42] and the fique fabric, as further discussed in the corresponding thermogravimetric curves. Precaution was taken that the samples would not touch each other or the inner walls. Table 1 lists the nomenclatures used for the samples in this work.

- 0 h (no aging): control sample;
- Aging temperature: 170 °C (T); and
- Aging time: 72, 120, 240 h.

The mass and dimensions, particularly the thickness, of the fique fabric–epoxy composite samples were measured before and after aging using a 0.001-g precision Gehaka scale and a 0.01-mm Mitutoyo digital caliper, respectively.

### 2.4. Experimental Procedures

#### 2.4.1. Scanning Electron Microscopy (SEM) Analysis

The microstructural properties, damage mechanisms, and degradation were monitored using scanning electron microscopy (SEM) images of the plain epoxy and fique fabric composite, in aged and unaged states. A model Quanta FEG250 FEI microscope Thermofisher Scientific, Hillsboro, OR, USA, operating with secondary electrons at 28 kV was used. Samples were gold-sputtered for electron conduction. The study focused on the accelerated aging of the surface layer of the composite and the plain epoxy, which means no “in core” analysis was intended.

#### 2.4.2. Thermogravimetric Analysis (TGA)

TGA curves of epoxy and fique fabric composites were carried out in a Shimadzu equipment, Tokyo, Japan. Samples were crushed and put in a platinum crucible to be analyzed under nitrogen atmosphere in a temperature range from 20 °C to 700 °C at a heating rate of 10 °C/min.

#### 2.4.3. Dynamic Mechanical Analysis (DMA)

DMA was used to characterize the temperature-dependent viscoelastic properties of the materials described in Table 1. The tests were performed using a dynamic mechanical analyzer DMA Q800 from TA Instruments (New Castle, DE, USA) operating at a frequency of 1 Hz with a heating rate of 3 °C/min under a nitrogen atmosphere. A three-point bending mode was used for all the samples. Curves of storage modulus (E’), loss modulus (E”), and tan delta (tan δ) in the temperature range from 30 °C to 190 °C were recorded just for the first run. 

## 3. Results and Discussions

In the present investigation, the effects of thermal aging on the morphology and thermo-viscoelastic-mechanical properties were evaluated at 170 °C for different exposure times.

### 3.1. Mass and Thickness Variations

**Figure 2:**
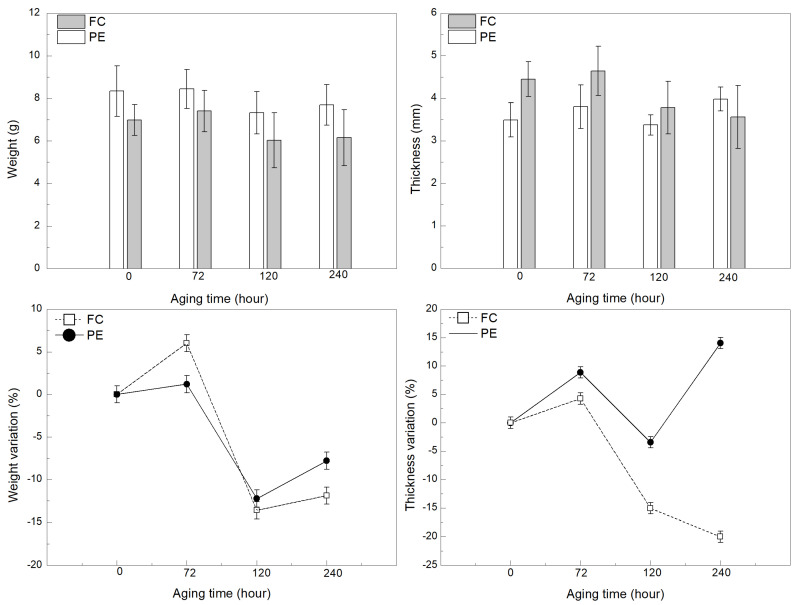
Mass and thickness losses of the plain epoxy (PE) and fique fabric reinforcing epoxy composite (FC) during thermal aging.

Figure 2 shows the mass and thickness variations in the plain epoxy and fique fabric composite caused by accelerated aging. In this figure, one can notice a mass increase of 6% and 1% for the composite (FC-T72) and plain epoxy (PE-T72) at 72 h of exposure time, respectively. Hinkley et al. [43] reported that it is common to verify this slight initial weight gain given by the formation of oxidation products. By contrast, a higher mass loss of 14% (FC-T120) and 13% (PE-T120) were observed in the samples exposed for 120 h. Additionally, after aging for 240 h, the FC-T240 and PE-T240 also presented a mass loss of 12% and 8%, respectively. This accelerated weight loss (>10%) might indicate the material embrittlement and occurrence of cracks, which increase the surface area of contact with oxygen [43,44]. Based on this result, regarding the mass loss, the fique fabric composite exposed for 120 h (FC-T120) was the most unstable condition. Furthermore, a high shrinkage of 15% was also displayed by the FC-T120 composite, which might be associated with the outgassing of resin oxidation by-products [45].

### 3.2. Effects of Thermal Aging on Fique Fabric

**Figure 3:**
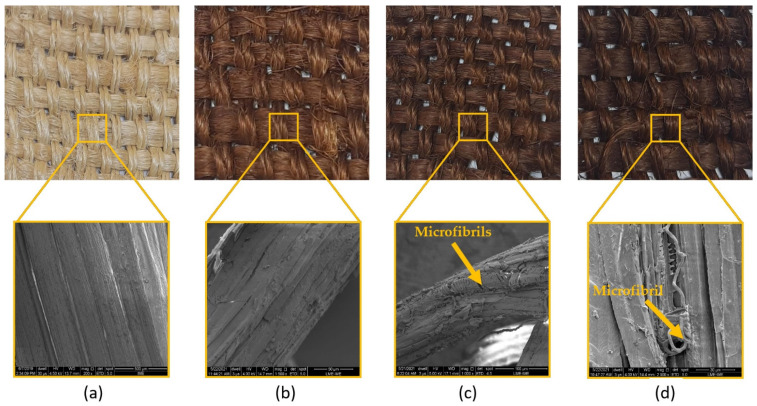
Visual changes and SEM micrographs of fique fiber after high temperature aging: (**a**) 0 h; (**b**) 72 h; (**c**) 120 h; (**d**) 240 h at high temperature (170 °C).

**Figure 4:**
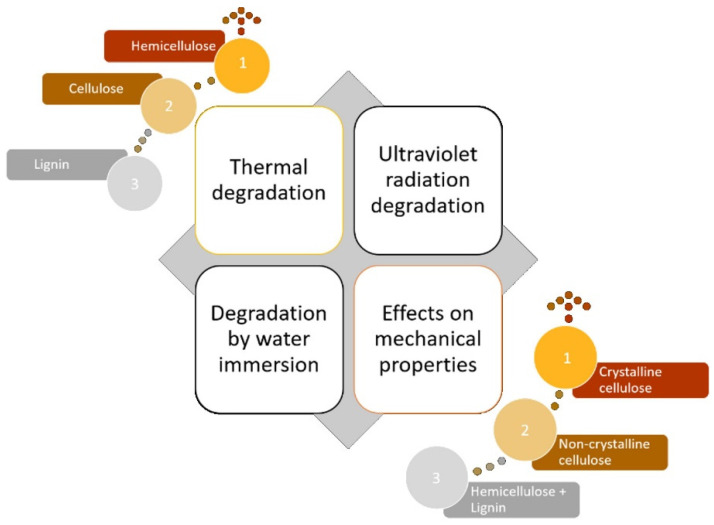
Main components of natural fibers and their orderly influence for thermal degradation and mechanical properties.

Figure 3 shows the SEM images of fique fiber unaged and aged with different exposure times. Microcracks can be noted along the fiber length exposed for 72 h (Figure 3b). The effect of thermal degradation was more evident after aging for 120 h (Figure 3c), for which we observed microfibrils exposed in the fique fiber surface. In Figure 3d, it is also noted the microfibrils’ breakage after 240 h of exposure. Collapsed microfibrils in heat-treated kenaf fibers were observed by Azwa and Yousif [46] and also reported by Ezekiel et al. [47] for coir fibers. The authors suggested that the presence of these collapsed microfibrils might be associated with the depolymerization of hemicellulose from 180 °C and can also contribute to the weight loss of the composites. Hence, the thermal stability was enhanced with an increase in hemicellulose content, as well as with a higher crystalline cellulose amount. Conversely, an increase in extractive content led to a reduction in thermal stability [29]. In other words, the initial thermal degradation started with hemicellulose degradation, as shown in Figure 4, which presented the scheme of the relationship between NLF components and thermal aging. Therefore, based on Figure 3, hemicellulose loss and oxidation might be the major mechanisms for the microstructure changes on the fique fiber exposed to thermal aging.

### 3.3. Effects of Thermal Aging on Plain Epoxy 

**Figure 5:**
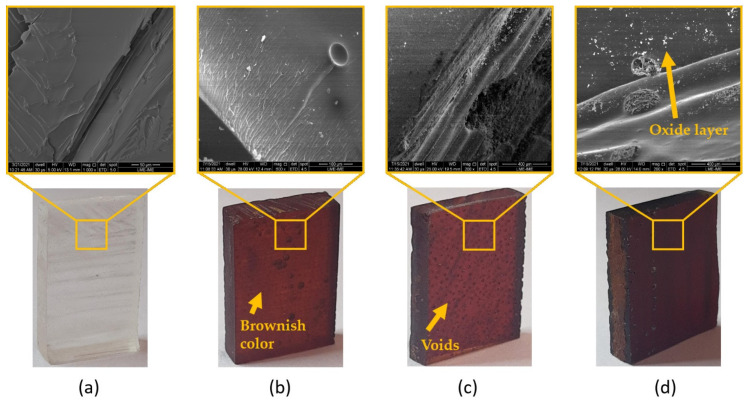
Visual changes and SEM micrographs of plain epoxy thermally aged in (**a**) 0 h; (**b**) 72 h; (**c**) 120 h; and (**d**) 240 h exposure time.

It is well known that epoxy properties are strongly dependent on the usage conditions, such as time and temperature. When exposed to temperatures of about 100–200 °C, polymers suffer from softening, creep, distortion, and deterioration of mechanical properties [48]. Figure 5 shows the occurrence of voids formed since the first exposure time, as well as the change of color, which is related to the irreversible aging mechanism. The oxidative evolution of carbonyl groups in the epoxy resin was the reason for the surface brownish color. In addition, microcracks could be observed in some aged samples.

The aging of polymers in an oxidative environment, such as the one employed in this study, leads to a heterogeneous degradation, which is governed by the diffusion of oxygen through the polymer [41]. Previous studies [41,48,49] have indicated that thermal aging has a strong influence on the properties of polymer-based materials. The white-colored spots in the SEM image of Figure 5d are related to an oxide layer observed in all thermally aged epoxies [50]. In addition, one should note that the voids act as reservoirs of them [51].

### 3.4. Effects of Thermal Aging on Fique Fabric-Reinforced Epoxy Composite

**Figure 6:**
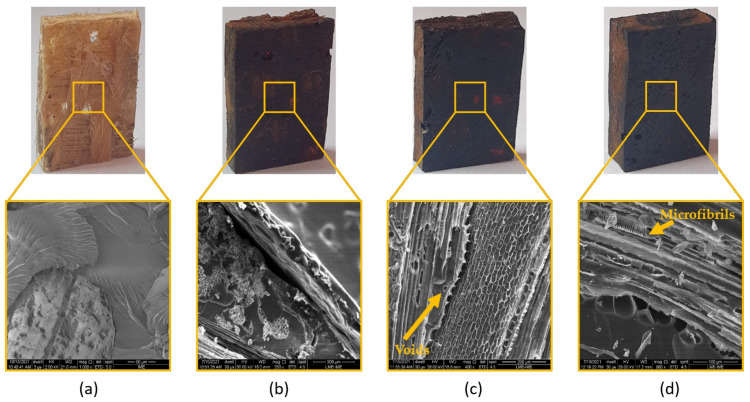
Visual changes and corresponding high magnification SEM micrographs of fique fabric-reinforced epoxy composites thermally aged in (**a**) 0 h; (**b**) 72 h; (**c**) 120 h; and (**d**) 240 h exposure time.

SEM images of fique fabric-reinforced epoxy composite unaged and thermally aged are shown in Figure 6. In this figure, it can be observed that the exposure to thermal aging increased the damage on fique fabric-reinforced epoxy composites through fiber pullout, fiber breakage, tearing, debris, and matrix cracking [52]. 

Thermal oxidation of the fique fabric–epoxy composite introduced damage at the fiber–matrix interfaces, which caused the formation of microcracks and degraded the structural property. It was possible to note that all exposure times promoted the fiber–matrix debonding and fiber fibrillation due to the degradation of less stable components of the fique fibers as well as shrinkage of the epoxy. The induced thermal stress may be relieved by crack formation in the matrix and fiber failure (Figure 3). Both matrix cracking and fiber failure degrade the mechanical properties of the composite. 

All samples of the fique fabric, plain epoxy, and fique fabric composites revealed modification in the macrostructural level by the color change. A brownish layer formation at the sample surface was also reported in the literature [46,49,53,54].

### 3.5. TGA of Unaged Epoxy and Composite

**Figure 7:**
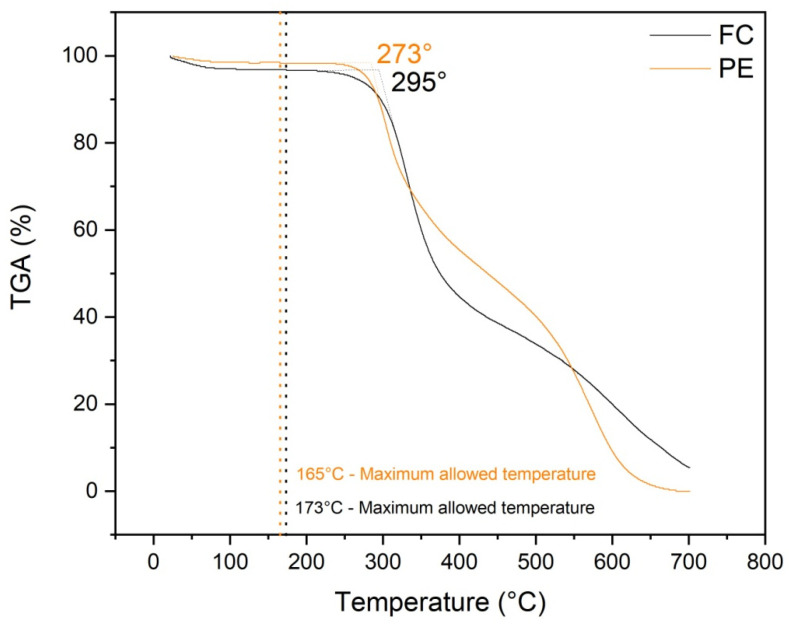
TGA curves of plain epoxy and unaged fique fabric composites.

Figure 7 shows thermogravimetric curves for plain epoxy and unaged fique fabric composites. These curves revealed that 170 °C might be considered the maximum temperature before a sudden decrease in mass loss associated with effective thermal degradation of the material. These TGA results justify the choice of 170 °C for the basic temperature for different aging times selected in the present work.

### 3.6. Effects of Thermal Aging on DMA

The dynamic mechanical relaxation behavior of fique fabric-reinforced epoxy composites and plain epoxy annealed at 170 °C up to 240 h and unaged was investigated. The main DMA parameters for both aged and unaged 40 vol% fique fabric composite and plain epoxy are shown in Table 2 and Table 3, respectively. Each of these parameters is further discussed in the subsections below.

#### 3.6.1. Storage Modulus

**Figure 8:**
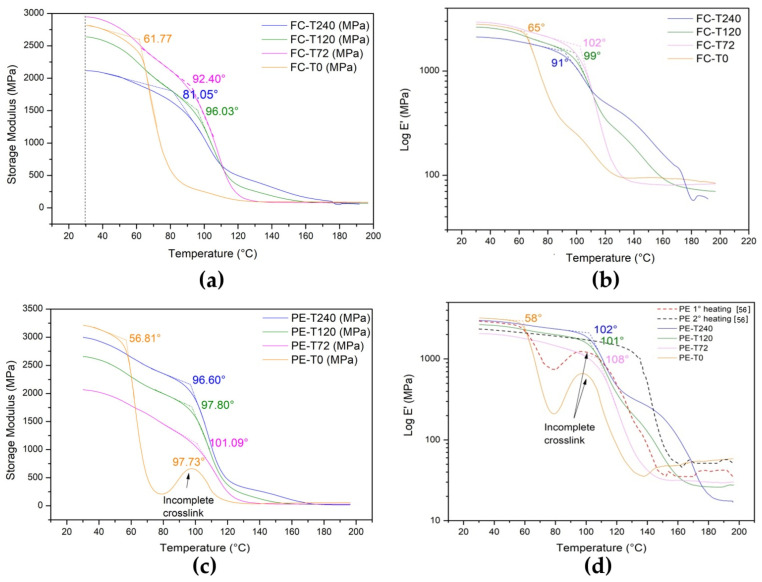
Storage modulus (E’) curves for samples unaged and thermally aged at different conditions: (**a**) fique fabric epoxy composites; (**b**) log E’ versus temperature for fique fabric epoxy composite; (**c**) plain epoxy; and (**d**) log E’ versus temperature for plain epoxy with additional first and second runs, dotted curves of the same DGEBA/TETA epoxy adopted from [55].

Figure 8 presents the storage modulus (E’) for both fique fabric composites (Figure 8a) and plain epoxy (Figure 8c) samples aged at 170 °C in different exposure times and unaged ones. It was observed that exposure time had a strong influence on the relaxation behavior of these materials, which is associated with the glass transition temperature (Tg) from crystalline state to rubbery plateau [21]. For better identification of the glassy plateau in the plain epoxy (unaged and aged), see Figure 8a–d with storage modulus Y axis in log scale, respectively. Figure 8d also shows dotted curves of the same DGEBA/TETA epoxy corresponding to the first and second DMA runs, as reported by Margem et al. [55].

One should notice that the E’ value of the FC-T72 was the highest among the conditions due to an increase of the chain mobility during the glassy/rubbery transition [10]. By contrast, the storage modulus decreased for 240 h aging (FC-T240), which was associated with the decrease in the material viscoelastic stiffness [56], indicating the degradation at the fiber–matrix interface, as verified in the SEM analyses. Additionally, it is worth mentioning the presence of a broad peak around 98 °C for the unaged plain epoxy. This can be attributed to the post-curing reaction given by the incomplete crosslinking. This peak in the value of E’ occurred in the first run of the DMA test, which corresponded to the experimental procedure in the present work. Indeed, a previous work [56] using the same DGEBA/TETA epoxy matrix reinforced with ramie fibers reported a similar peak around 100 °C that disappeared in the second run. Such an E’ peak was attributed to an additional crosslink completion process. Stark [57] also reported an E’ peak behind 175 °C in a type 6376 epoxy from Hexcel, for application in aircraft carbon–epoxy composites. Similarly, the author indicated that crosslinking would be the expected consequence of this peak. 

Furthermore, the results indicated a more pronounced effect of exposure time on aged composite samples in comparison to plain epoxy ones. It might have been caused by the fiber–matrix interface weakening on the composites. Nevertheless, for both fique fabric composites and plain epoxy, an increase of Tg was observed for the samples aged up to 120 h. The results revealed an increase of up to 45 °C on Tg, the same as found by Inamdar et al. [41].

Another point worth noting in Figure 8d is the onset of the sudden drop in the value of E’ after a glassy plateau, which extended well under room temperature in the work of Stark [57]. The author mentioned that crosslinking-induced vitrification should lead to a plateau in E’. Aging at 170 °C significantly extended the onset of the sudden drop to temperatures above 100 °C, which might also be attributed to crosslinking triggered by heating above T_g_. This provided the thermal energy to activate monomers to further crosslink and then the storage modulus achieved a plateau associated with final crosslinking reactions [58].

#### 3.6.2. Loss Modulus

**Figure 9:**
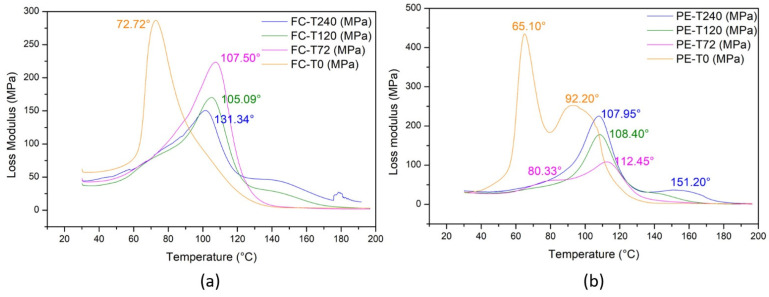
Loss modulus (E”) vs. temperature with glass transition temperatures (Tg) highlighted for samples unaged and thermally aged at different conditions: (**a**) fique fabric-reinforced epoxy composites; (**b**) plain epoxy.

It is well known that another noticeable effect of the 3D molecular arrangement of the polymer matrix is the main peak in the curve of loss modulus (E”). Figure 9 shows the loss modulus (E”) vs. temperature for the fique fabric composite and plain epoxy at different times of aging. The maximum value of E” was attributed to the relaxation alpha (α) peak [20], which was associated with the mobility of the polymer chains in the transition from crystalline to an amorphous molecular structure. In this figure, one should notice that the α-peaks are displaced to higher temperatures in comparison to plain epoxy. This indicates a decrease in the flexibility of the epoxy chains caused by the incorporation of fique fabric. Additionally, the broadening of this peak for higher exposure times denotes the evolution of the macromolecular network at the fiber–matrix interface and debonding during aging, modifying the relaxation processes associated with the α-transition [59].

#### 3.6.3. Tan Delta

**Figure 10:**
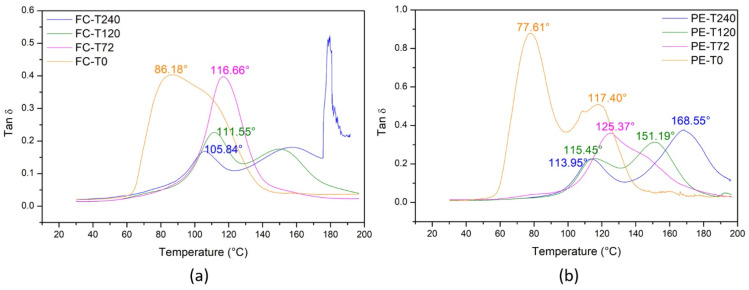
Effect of time aging on the loss tangent (tan δ) curves for: (**a**) fique fabric-reinforced epoxy composites; and (**b**) plain epoxy.

The loss factor (tan δ) is the E”/E’ ratio, which represents the mechanical damping or molecular internal friction of a viscoelastic configuration. The effect of time aging on the tan δ for the fique fabric-reinforced epoxy composite and plain epoxy is shown in Figure 10. The tan δ curves show an expected peak shift to lower temperatures after aging. An apparent increase in peak intensity is observed for FC-T72, indicating higher mobility of the macromolecular chains [60], which corroborates the analysis performed for E’ in Figure 8. For samples aged at higher times, the trend of broadening and the decrease of tan δ peaks suggests the weakness of the material [10]. Furthermore, the heterogeneity of the macromolecular networks with the formation of new crosslinked domains is confirmed by extra peaks in FC-T240 (Figure 10a).

#### 3.6.4. Modeling of the Loss Factor Curve: Evaluation with Exponentially Modified Gauss (EMG) Distribution Functions

**Figure 11:**
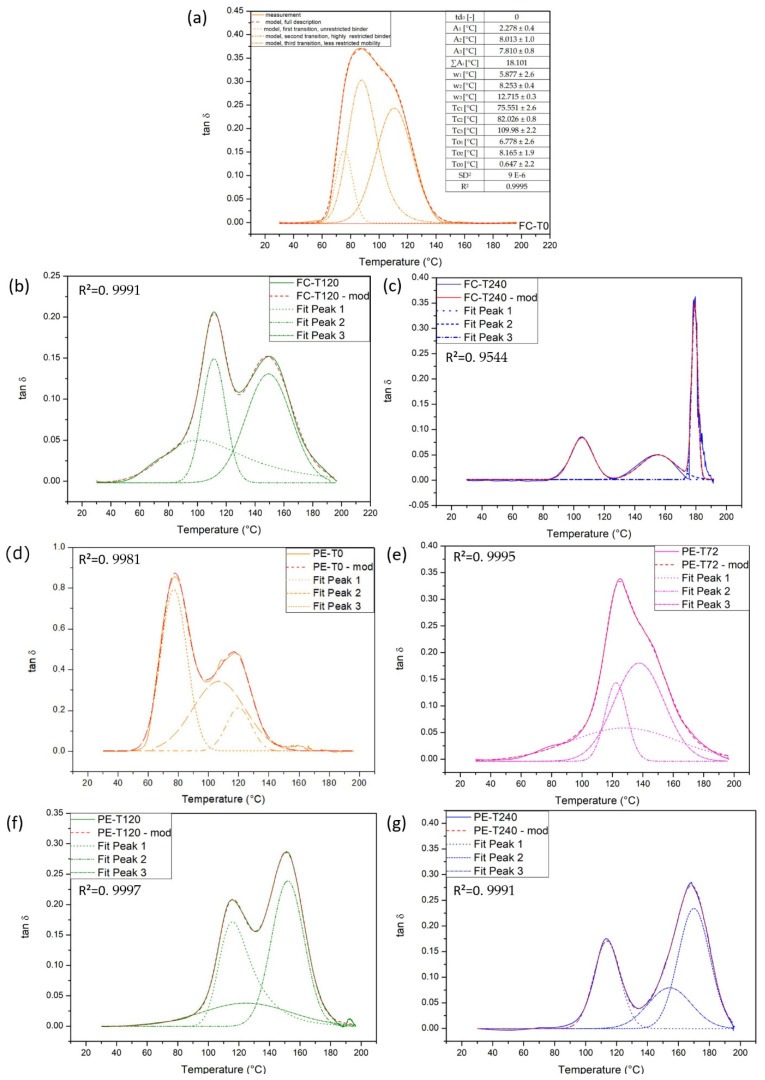
Description of loss factors of the plain epoxy and fique fabric composites in different aging conditions with three EMG functions. (The solid curves represent the original data and the dashed lines are relative to the result after multi-peak resolution.) (**a**) FC-T0; (**b**) FC-T120; (**c**) FC-T240; (**d**) PE-T0; (**e**) PE-T72; (**f**) PE-T120; and (**g**) PE-T240.

DMA investigations revealed distinct changes in the shape of the loss factor (tan δ) curve. One can note that the tan δ curves, in Figure 10, contain two apparent peak regions, i.e., exhibit at least two mobility fractions. The molecular interpretation of the involved aging phenomena on the tan δ curves was investigated by modeling with three exponentially modified Gauss (EMG) distribution functions. This model consisted of a convolution between Gauss distribution and exponential decay function, which was described in detail elsewhere [8]. The application of EMGs provided parameters of the sub-transition regions of the loss factor curve: (1) the peak areas (A_i_); (2) the half peak width at half height of the Gaussian part of corresponding EMG (w_i_); (3) the temperature at peak maximum of Gaussian part of the EMG (Tc_1_); and (4) the relaxation parameter in the exponential part of the EMG (To_i_) [7,8,9]. Figure 11 shows the loss factor description for fique fabric composite and plain epoxy with three EMGs. In this figure, the deconvoluted peaks for the different regions of molecular rearrangement processes correspond to the polymer fractions with different mobility [7,8,9]. After the baseline correction, a suitable function was selected to evaluate the area under the peaks. It can be noted that all graphs obtained a high R^2^ coefficient. The number of fit parameters obtained was equal to N×4, where N is the number of EMGs’ fit functions and 4 means the number of parameters per EMG (A_i_, w_i_, Tc_i_, To_i_). 

Table 4 shows the parameters obtained from the EMG curves (Figure 11). This table reveals that almost all parameters were aging dependent, mainly in the second transition (peak 2), where its asymmetry decreased and its intensity increased. It was also noted that for all transitions of plain epoxy, the maximum temperatures of the Gauss parts of the EMGs (Tc_1_, Tc_2_, and Tc_3_) increased with aging time. The same process was considered for fique fabric composite, and the EMGs’ fit parameters are presented in Table 5.

The sum of peak areas is considered the total area of the loss factor curve, and for almost all conditions for both plain epoxy and fique fabric composites these values decreased. This means that restrictions on chains’ mobility increased. Furthermore, a small peak area (A_i_) was caused by (1) hindrance in mobility, (2) increase of stiffness, and (3) increase of crosslinking. In addition, the peak temperature (Tc_i_) of the Gauss part can be interpreted as Tg of the relaxation free transition, i.e., transition without the exponential part; one has a pure Gauss distribution. By contrast, when the exponential relaxation (To_i_) was more pronounced, the higher was the residual internal friction.

Based on Table 4 and Table 5, as the molecular mobility increased, the area under the tan δ curve and the amplitude of tan δ peak (Figure 11) increased. Higher tan δ means better damping (dissipation of energy) ability. Hence, if a structure that needs to absorb more dynamic energy is desired, the area under the tan δ curve should be increased. Regarding the amplitudes of tan δ curves in Figure 11, the results support that high-temperature aging has lower tan δ peaks, which means a worse damping capability. In polymer composites, the fiber reinforcement and the interfacial interaction influence the damping factor, which provides a synergy effect between viscous and elastic phases in the material. Comparing the values obtained for the fique fabric composite and the plain epoxy, it can be observed that the lower tan δ of the composite reflects good load-bearing properties of the system.

Similar behavior of peak 2 was found for the fique fabric-reinforced epoxy composite in comparison to plain epoxy. In both situations, what could have occurred what the literature reports as a diffusion-limited oxidation (DLO) phenomenon. The DLO facilitates the formation of a heterogeneous oxidized layer such that the regions near the air-exposed surfaces are heavily oxidized compared to the interior regions, which show reduced or minimal oxidation [41]. In summary, the DLO of polymers is the combination of two processes: (1) diffusion of oxygen and (2) reaction of oxygen. Atmospheric oxygen diffuses into the porous network of a polymeric material through its exposed surfaces, and the diffused oxygen reacts to form an oxide compound. DLO is more critical at elevated temperatures because the rate has higher activation energies than the permeability. It is worth mentioning that Pei et al. [42] also observed a double transition for the unaged control group, where the first peak was related to the glass–rubber transition of the material interior, and the second, the material exterior. The quantification of the effects, interaction, and amount of each aging time condition on the loss factor curve was possible using a model based on EMG distribution functions.

From these results it is proposed that the thermal degradation behavior of natural fibers, especially fique fiber, and their composites might be assessed, based on their structural characteristics and mechanical properties.

## 4. Summary and Conclusions

- Hemicellulose loss and oxidation are believed to be the main mechanisms for the microstructure changes that occur during thermal aging of fique fabric-reinforced epoxy composites. All samples of fique fabric, plain epoxy, and fique fabric composites revealed physical aspect changes at the macrostructural level, exhibiting a formation of a brownish layer at the sample surface.
- Mass loss rapidly increased at the initial stage of thermal aging, mainly due to the evaporation of moisture and the volatilization of residual low-molecular-weight substances. The samples thermally exposed for 120 h showed the higher mass loss, ∼14%, and high shrinkage, ~15%, which might be associated with the outgassing of resin oxidation by-products.
- In DMA analyses, we observed significant property changes after aging. The most notable changes occurred in the storage modulus (E’) curves. For samples aged for 72 h, a significant decrease in E’ modulus was observed for both the plain epoxy and the fique fabric composite. In addition, an increase of Tg was observed for the samples aged up to 120 h.
- By contrast, the fique fabric composite aged with 240-h aging time exhibited a lower E’ modulus, which indicated the degradation at the fiber–matrix interface, also verified in the SEM analyses.
- Additionally, it is worth mentioning the presence of a broad peak around 98 °C for the unaged plain epoxy, which was attributed to the post-curing reaction given by the incomplete crosslinking.
- The loss factor results were modeled with three exponentially modified Gauss functions in order to get a molecular interpretation of the involved phenomena, which can be analyzed by the areas of the second and third transition peaks or the corresponding temperature values of the assigned Gauss peaks. The fique fabric composites presented smaller peak areas compared to the plain epoxy, which might be explained by the higher hindrance in mobility, an increase of stiffness, and an increase of crosslinking.

## Figures and Tables

**Table 1:** Nomenclature adopted for DMA specimens.

Material	Aging Times (Hours at 170° C)			
	0	72	120	240
Plain epoxy	PE-T0	PE-T72	PE-T120	PE-T240
Composite	FC-T0	FC-T72	FC-T120	FC-T240

**Table 2:** DMA parameters obtained for the composite reinforced with 40 vol% fique fabric, aged at high temperature and unaged.

Material	E’_30_		E”		Tan δ	
	Storage Modulus at 30 °C (GPa)	Lower Limit of Tg (°C)	Loss Modulus Peak (GPa)	Peak Temperature (°C)	Tan δ Peak (GPa)	Peak Temperature (°C)
FC-T0	2.81	61.77	0.29	72.95	0.40	86.82
FC-T72	2.94	92.40	0.22	107.55	0.40	116.71
FC-T120	2.64	96.03	0.17	105.14	0.18	150.19
FC-T240	2.12	81.05	0.15	101.39	0.58	179.71

**Table 3:** DMA parameters obtained for plain epoxy, aged at high temperature and unaged.

Material	E’_glassy(30 °C)_		E”		Tan δ	
	Storage Modulus at 30°C (GPa)	Lower Limit of Tg (°C)	Loss Modulus Peak (GPa)	Peak Temperature (°C)	Tan δ Peak (GPa)	Peak Temperature (°C)
PE-T0	3.21	97.97	0.25	93.61	0.51	117.81
PE-T72	2.07	102.37	0.11	112.86	0.24	145.29
PE-T120	2.65	99.82	0.28	142.88	0.31	151.29
PE-T240	2.99	97.04	0.37	151.88	0.38	169.13

**Table 4:** EMG fit parameters of the loss factor curves for plain epoxy as a function of aging at high temperature. The column named ‘change’ gives the ratio of values between the aged state at 170 °C, 240 h, and the unaged state.

	Plain Epoxy					
Aging Time	0	72	120	240	Change	Trend with Aging
A_1_	17.239 ± 0.1	5.538 ± 0.2	5.157 ± 0.1	3.647 ± 0.0	0.21	decrease
A_2_	4.379 ± 0.1	2.661 ± 0.1	6.393 ± 0.0	6.011 ± 1.0	1.37	increase
A_3_	14.874 ± 0.2	7.596 ± 0.3	2.785 ± 0.1	2.791 ± 1.1	0.19	decrease
∑A_i_	36.492	15.795	14.335	12.449	0.34	-
w_1_	8.691 ± 0.0	34.950 ± 3.1	6.857 ± 0.0	8.500 ± 0.2	0.98	nearly constant
w_2_	8.230 ± 0.5	7.190 ±0.1	10.645 ± 0.0	10.212 ±0.3	1.24	increase
w_3_	17.356 ± 0.0	16.442 ± 5.1	29.004 ± 0.4	13.963 ± 3.5	0.80	nearly constant
Tc_1_	77.095 ± 0.1	122.156 ± 22.3	108.58 ± 0.1	113.10 ± 5.2	1.47	increase
Tc_2_	119.28 ± 8.4	122.20 ± 0.0	152.00 ± 0.1	169.52 ± 0.4	1.42	increase
Tc_3_	106.13 ± 0.2	137.42 ± 0.4	124.80 ± 2.46	153.57 ± 1.2	1.45	increase
To_1_	3.18 × 10^−12^ ± 0.1	5.993 ± 0.2	14.800 ± 0.3	0.733 ± 0.5	-	decrease
To_2_	0.793 ± 0.8	0.181 ± 0.0	4.5 × 10^−12^ ± 0.0	0.509 ± 0.3	0.64	decrease
To_3_	0.790 ± 0.0	0.164 ± 0.4	8.5 × 10^−11^ ± 0.0	0.770 ± 0.0	0.97	nearly constant
R^2^	0.9981	0.9995	0.9997	0.99905	-	-

**Table 5:** EMG fit parameters of the loss factor curves for fique fabric-reinforced epoxy composite as a function of aging at high temperature. The column named ‘change’ gives the ratio of values between the aged state at 170 °C, 240 h, and the unaged state.

	Fique Fabric Reinforcing Epoxy Composite (°C)				
Aging Time	0	120	240	Changes	Trend with Aging
A_1_	2.278 ± 0.4	4.226 ± 0.0	1.483 ± 0.0	0.65	decrease
A_2_	8.013 ± 1.0	3.097 ± 0.0	1.408 ± 0.0	0.18	decrease
A_3_	7.810 ± 0.8	5.220 ± 0.0	1.716 ± 0.0	0.22	decrease
∑A_i_	18.101	12.543	4.607	0.25	-
w_1_	5.877 ± 2.6	19.017 ± 0.4	7.175 ± 0.2	1.22	increase
w_2_	8.253 ± 0.4	8.182 ± 0.0	11.475 ± 0.4	1.39	increase
w_3_	12.715 ± 0.3	15.686 ± 0.0	1.984 ± 0.0	0.16	decrease
Tc_1_	75.551 ± 2.6	80.883 ± 0.7	105.42 ± 0.1	1.40	increase
Tc_2_	82.026 ± 0.8	111.46 ± 0.0	155.20 ± 0.3	1.89	increase
Tc_3_	109.98 ± 2.2	148.97 ± 0.1	179.20 ± 0.0	1.63	increase
To_1_	6.78 × 10^−11^ ± 2.6	39.103 ± 1.2	4.65 × 10^−18^ ± 0.0	-	increase
To_2_	8.165 ± 1.9	3.56 × 10^−13^ ± 0.0	2.35 × 10^−17^± 0.0	-	decrease
To_3_	0.647 ± 2.2	0.411 ± 0.0	4.15 × 10^−15^ ± 0.0	-	increase
R^2^	0.9995	0.9991	0.9544	-	-

## Data Availability

Not applicable.
